# Feasibility of a break-in period of less than 24 hours for urgent start peritoneal dialysis: a multicenter study

**DOI:** 10.1080/0886022X.2022.2049306

**Published:** 2022-03-10

**Authors:** Xi Wen, Liming Yang, Zhanshan Sun, Xiaoxuan Zhang, Xueyan Zhu, Wenhua Zhou, Xiaoqing Hu, Shichen Liu, Ping Luo, Wenpeng Cui

**Affiliations:** a Division of Nephrology, The Second Hospital of Jilin University, Changchun, China; b Division of Nephrology, The First Hospital of Jilin University-the Eastern Division, Changchun, China; c Division of Nephrology, Xing’anmeng people’s Hospital, Ulan Hot, China; d Division of Nephrology, Jilin FAW General Hospital, Changchun, China; e Division of Nephrology, Jilin City Central Hospital, Jilin, China

**Keywords:** Urgent start peritoneal dialysis, peritoneal dialysis, break-in period, complications, technique failure

## Abstract

**Purpose:**

Urgent start peritoneal dialysis (USPD) is an effective therapeutic method for end-stage renal disease (ESRD). However, whether it is safe to initiate peritoneal dialysis (PD) within 24 h unclear. We examined the short-term outcomes of a break-in period (BI) of 24 h for patients undergoing USPD.

**Methods:**

This real-world, multicenter, retrospective cohort study evaluated USPD patients from five centers from January 2013 to August 2020. Patients were divided into BI ≤ 24 h or BI > 24 h groups. The Primary outcomes included incidence of mechanical and infectious complications. The secondary outcome was technique failure. Moreover, we presented a subgroup analysis for patients who did not receive temporary hemodialysis (HD).

**Results:**

A total of 871 USPD patients were included: 470 in the BI ≤ 24 h and 401 in the BI > 24 h groups. Mechanical and infectious complications did not differ between the two groups across the follow-up timepoints (2 weeks, 1 month, 3 months, and 6 months) (*p* > 0.05). Multiple logistic regression analysis revealed that BI ≤ 24 h was not an independent risk factor for mechanical complications, catheter migration, or infectious complications (*p* > 0.05). A BI ≤ 24 h was not an independent significant risk factor for technique failure by multivariate Cox regression analysis (*p* > 0.05). The subgroup analysis of patients who did not receive temporary HD returned the same results.

**Conclusion:**

Initiating PD within 24 h of catheter insertion was not associated with increased mechanical complications, infectious complications, or technique failures.

## Introduction

End-stage renal disease (ESRD) is a chronic condition in many countries [1] and patients with ESRD often require urgent renal replacement treatment [2,3]. Currently, unplanned dialysis is common among patients with ESRD who may experience unexpected deterioration of renal function [4,5]. As an unplanned dialysis modality, urgent start peritoneal dialysis (USPD) is as effective as urgent start hemodialysis (USHD), and might be more appropriate for patients with poor economic conditions, poor vascular networks that limit hemodialysis (HD), and promising preservation of residual renal function [6–9].

Urgent start peritoneal dialysis is defined as initiating peritoneal dialysis (PD) less than 14 days after catheter insertion [10]. Although a number of studies have documented that USPD is a safe and effective therapeutic modality for patients with ESRD compared to conventional-start PD [11–14], the 14 day break-in period (BI) for USPD is too long for patients with severe uremic symptoms [15]. The optimal BI for USPD remains unclear and the results of studies focusing on this have been inconsistent. For example, several studies report that BIs of 2 days [16] and 7 days [17] may increase the incidence of catheter-related complications, while other studies with small sample sizes indicate that BIs of 2 days [12] and 3 days [18] did not increase catheter-related complications. These contradictory results may be a result of variations in trial design, surgical modality, and geography.

Several studies evaluating the feasibility of USPD with a shorter BI, found that a BI < 24 h increased the incidence of mechanical complications [19,20]. However, there were several limitations to these studies. Some were single-arm studies [19–21]. Another study compared a BI < 24 h with a BI > 7 days for complications, but the surgical modality was heterogenous between groups; therefore, it is possible that the surgical modality impacted on the results [22]. The other study only investigated the influence of initiating automated peritoneal dialysis (APD) within 24 h of catheter insertion [23]. To further clarify whether a BI ≤ 24 h was feasible and to provide guidance for clinical use, we performed a multicenter, retrospective cohort study to compare the incidence of short-term complications and technique failure between patients with a BI ≤ 24 h and a BI > 24 h. We also investigated whether a BI ≤ 24 h was a risk factor for short-term complications and technique failure.

## Materials and methods

### Study design and patients

Patients who required PD for ESRD, diagnosed between 1 January 2013, and 31 August 2020, were eligible for our retrospective study. Subjects were recruited from The Second Hospital of Jilin University; The First Hospital of Jilin University-the Eastern Division; Jilin FAW General Hospital; Jilin City Central Hospital; and Xing’anmeng People’s Hospital. Some patients required temporary HD treatment before and/or after PD for severe uremic symptoms. The inclusion criteria were: (1) the patients were diagnosed with ESRD; (2) PD was initiated between 1 January 2013, and 31 August 2020. The exclusion criteria were (1) patients with incomplete data, (2) non-USPD patients, (3) patients younger than 18 years, (4) patients treated with Chronic HD, which was defined as enrollment in an HD program for more than 3 months and undergoing more than seven HD sessions monthly [24], and (5) patients who underwent percutaneous catheter surgery or laparoscopic surgery.

The study design was approved by the Ethics Committee of the Second Hospital of Jilin University with study ID (No. 2020031, retrospectively registered). Due to the retrospective study design, informed consent was not required. The research was conducted in compliance with the Declaration of Helsinki.

All patients received the same routine preoperative preparation, which included skin preparation, prophylactic antibiotic administration, and emptying of the bowel and bladder. Prophylactic preoperative antibiotics were given according to International Peritoneal Dialysis Association and the antibiotics were determined by the local spectrum of antibiotic resistance (usually penicillin or cephalosporins) [25,26]. Patients received open surgery with a polyester double cuff straight Tenckhoff catheter, placed by the same group of experienced clinicians in all cases. The number of clinicians who performed PD catheter implantation included four in The Second Hospital of Jilin University, two in The First Hospital of Jilin University-the Eastern Division, one in Jilin FAW General Hospital, two in Jilin City Central Hospital, and one in Xing’anmeng People’s Hospital. All the clinicians had completed professional medical training regarding the process of catheter insertion as previously described [27]. In addition, peritoneal irrigation was performed with peritoneal dialysate after catheter placement. Patients were divided into the BI ≤ 24 h group and the BI > 24 h group according to the BI, which was determined according to the clinical situation of each patient by their nephrologists.

Peritoneal dialysis was initiated as APD or continuous ambulatory PD (CAPD) in this study. Peritoneal dialysis was prescribed based on fluid overload, uremia, hyperkalemia, and acid-base imbalance for each patient. Usually, a low-volume abdominal cavity (0.5–1.0 L) was initially assumed by the local treating team, and the volume was progressively increased to two L per cycle within 2 weeks. For CAPD patients, four cycles over 3–4 h were performed per day, while for APD patients, six-nine cycles over 48 min and an overnight dwell were performed per day. The dialysis procedure was performed by a PD nurse until the patient and/or caregiver could perform the process independently.

### Data collection

Data collection included (1) patient demographics: sex, age, combined temporary HD performed, dialysis mode during the BI period, cause of ESRD, comorbidities, history of abdominal surgery, date of catheter insertion, and date of PD initiation; (2) laboratory indicators (preoperative): white blood cells (WBC), hemoglobin, albumin, triglyceride, total cholesterol (TC), high-density lipoprotein (HDL), low-density lipoprotein (LDL), creatinine, uric acid (UA), blood urea nitrogen (BUN), estimated glomerular filtration rate (eGFR), K, Na, Ca, P, and blood glucose (BG); and (3) complications: date and incidence of mechanical (dialysate leakage, catheter migration, omental wrap, and hernia) and infectious complications.

### Clinical outcomes and definitions

The primary outcomes were the incidence of mechanical and infectious complications, such as dialysate leakage, catheter migration, omental wrap, hernia, and cases requiring surgical correction, at 2 weeks, 1 month, 3 months, and 6 months post-surgery. If mechanical and infectious complications occurred, conservative treatment was preferred, and surgical interventions were only performed when those measures were unsuccessful. Dialysate leakage was defined as dialysate flowing from the wound, the site of the incision, or through a weakness of the peritoneum, and was confirmed by visual observation, computed tomography, ultrasound, or the methylene blue method. Catheter migration was defined as the catheter tip being located outside the true pelvis and was confirmed by abdominal radiography and poor outflow [11]. Omental wrapping could be confirmed by secondary surgery. Hernia was defined as the intestine protruding from the incision or weakness of the abdominal wall. Infectious complications included exit-site infection, tunnel infection, and peritonitis. Exit site infection was diagnosed when purulent drainage, with or without erythema, was found at the exit site. A tunnel infection was diagnosed in the presence of clinical symptoms (such as erythema, edema, and/or tenderness) over the subcutaneous catheter pathway, associated with sanguinous, purulent, or thick drainage, either spontaneously or when pressure was applied to the catheter tunnel. Peritonitis was diagnosed when at least two of three criteria were met: (1) abdominal pain, (2) cloudy dialysis effluent with WBC count >100 cells/mm^3^ and 50% polymorphonuclear neutrophils, (3) positive culture from dialysate [28,29].

The secondary outcome was technique failure, which was defined as HD replacing PD for at least 1 month [30,31]. Temporary HD was defined as HD treatment within 3 months before and/or after PD initiation.

To avoid the impact of temporary HD on the results, we presented a separate subgroup analysis for the patients who did not receive temporary HD.

### Statistical analysis

All analyses were performed using SPSS Statistics 25.0. The measurement data were expressed as $\bar{X}$±S and *t*-tests were used for comparison among groups if the data conformed to a normal distribution, if not, data were expressed as M (1/4,3/4) and Wilcoxon rank-sum test was used for comparison among groups. The count data were presented as *n* (%), and comparisons among groups were performed using the chi-squared test or Fisher exact probability method. Factors associated with complications were analyzed using logistic regression analysis. Variables with *p* < 0.1 in the univariate analysis were included in the multivariate logistic regression analysis. The independent predictors of technique failure were identified by multivariate Cox modeling analysis, and only covariables with significance *p* < 0.1, were retained. Graphs were generated using GraphPad Prism software. Differences were considered statistically significant at *p* < 0.05.

## Results

### Demographic and clinical characteristics

**Figure 1.**
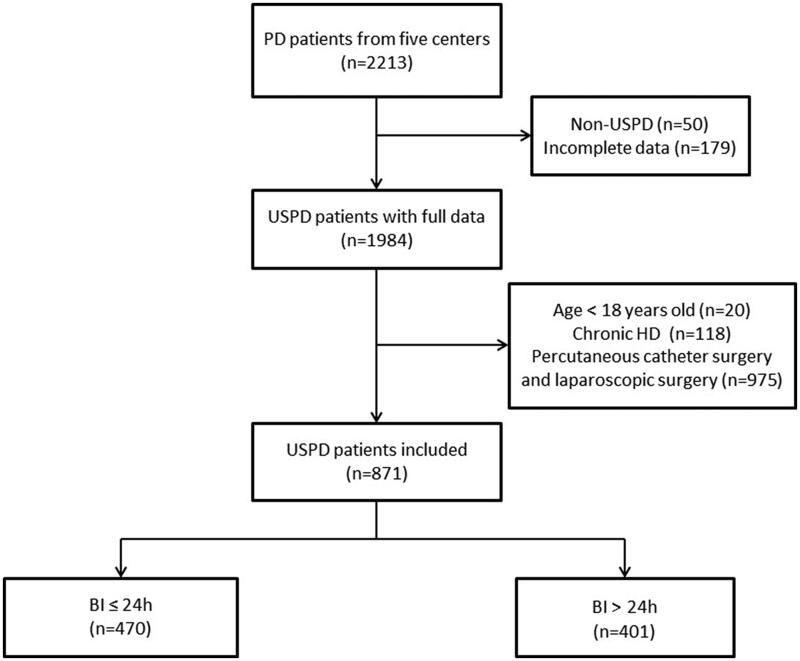
Flowchart. PD: peritoneal dialysis; USPD: urgent start PD; HD: hemodialysis; BI: break-in period.

A total of 2213 patients who received PD were screened, and 871 USPD patients from five centers were included in the study. Included patients were divided into the BI ≤ 24 h group (*n* = 470) and the BI > 24 h group (*n* = 401) (Figure 1). The mean initiation time of PD was 0.47 ± 0.50 days for the BI ≤ 24 h group and 4.26 ± 2.48 days for the BI > 24 h group. The baseline characteristics of the entire cohort and the two groups are shown in Table 1. The patients in the BI ≤ 24 h group were more likely to be male and younger (*p* < 0.05) than those in the BI > 24 h group. The cause of ESRD differed significantly between groups: the BI ≤ 24 h group was more likely to have hypertension as the cause of ESRD and less likely to have polycystic kidneys as the cause of ESRD than the BI > 24 h group (*p* < 0.05). The BI ≤ 24 h group had a lower incidence of temporary HD, APD and had lower WBC, triglyceride, and total cholesterol than the BI > 24 h group (*p* < 0.05). There were no statistically significant differences in the history of abdominal surgery or other laboratory indicators between the two groups (*p* > 0.05).

**Table 1.** Baseline characteristics of patients in different BI groups.

	Overall (*n* = 871)	BI ≤ 24h (*n* = 470)	BI > 24h (*n* = 401)	*X*^2^/*Z*-value	*p*-Value
Sex (male %)	518 (59.5%)	311 (66.2%)	207 (51.6%)	19.004	<0.001
Age (years)	53.00 (41.00,62.00)	51.00 (38.00,60.00)	55.00 (44.00,64.00)	−3.626	<0.001
Dialysis mode (APD %)	439 (50.4%)	209 (44.5%)	230 (57.4%)	14.379	<0.001
Temporary HD [*n* (%)]	277 (31.8%)	114 (24.3%)	163 (40.6%)	26.811	<0.001
Cause of ESRD [*n* (%)]				17.147	0.009
CGN	299 (34.3%)	158 (33.6%)	141 (35.2%)		
Diabetes	270 (31.0%)	138 (29.4%)	132 (32.9%)		
Hypertension	147 (16.9%)	93 (19.8%)	54 (13.5%)		
Interstitial nephritis	42 (4.8%)	18 (3.8%)	24 (6.0%)		
PKD	14 (1.6%)	3 (0.6%)	11 (2.7%)		
Others	49 (5.6%)	27 (5.7%)	22 (5.5%)		
Unknown cause	50 (5.7%)	33 (7.0%)	17 (4.2%)		
Comorbidities [*n* (%)]					
Hypertension	804 (92.3%)	434 (92.3%)	370 (92.3%)	0.002	0.969
Diabetes	310 (35.6%)	155 (33.0%)	155 (38.7%)	3.040	0.081
History of abdominal surgery [*n* (%)]	104 (11.9%)	50 (10.6%)	54 (13.5%)	1.646	0.200
Break-in period (days)	2.22 ± 2.56	0.47 ± 0.50	4.26 ± 2.48		
Laboratory indicators					
WBC (10*9/L)	6.49 (5.20, 8.04)	6.30 (5.10,7.90)	6.60 (5.33, 8.21)	−2.721	0.006
Hemoglobin (g/L)	85.00 (74.00,99.00)	86.00 (73.00,102.00)	85.00 (74.00, 96.50)	−1.632	0.103
Albumin (g/L)	34.00 (30.60,38.00)	34.00 (30.68,38.10)	33.90 (30.45, 37.95)	−0.347	0.728
TG (mmol/L)	1.66 (1.20,1.66)	1.66 (1.05,1.68)	1.66 (1.44,1.66)	−3.267	0.001
TC (mmol/L)	4.52 (3.98,4.71)	4.52 (3.79,4.79)	4.52 (4.30,4.67)	−2.223	0.026
HDL (mmol/L)	1.05 (0.91,1.08)	1.05 (0.89,1.13)	1.05 (0.93,1.05)	−0.336	0.737
LDL (mmol/L)	2.83 (2.34,2.85)	2.83 (2.31,2.91)	2.83 (2.40,2.83)	−1.389	0.165
Cr (µmol/L)	741.30 (573.40, 950.80)	739.50 (595.95, 976.80)	741.60 (554.25, 933.76)	−1.177	0.239
UA (µmol/L)	428.00 (355.00, 510.20)	428.00 (363.00, 521.00)	428.00 (344.00, 497.50)	−1.823	0.068
BUN (mmol/L)	21.56 (15.10, 28.90)	21.56 (14.33, 29.73)	21.56 (15.38, 28.32)	−0.099	0.921
eGFR	5.83 (4.36, 7.84)	5.83 (4.42, 7.83)	5.83 (4.29, 7.85)	−0.730	0.465
K (mmol/L)	4.38 (3.90, 4.94)	4.38 (3.90, 4.95)	4.38 (3.95, 4.94)	−0.635	0.525
Na (mmol/L)	140.20 (138.00, 142.30)	140.20 (138.20, 142.13)	140.20 (138.00, 142.70)	−0.122	0.903
Ca (mmol/L)	2.02 (1.87, 2.16)	2.02 (1.88, 2.18)	2.02 (1.86, 2.16)	−0.550	0.583
P (mmol/L)	1.74 (1.40, 2.16)	1.74 (1.40, 2.15)	1.74 (1.41, 2.18)	−0.039	0.969
BG (mmol/L)	5.20 (4.76, 5.95)	5.20 (4.72, 5.90)	5.20 (4.80, 6.07)	−0.809	0.418

BI: break-in period; APD: automated peritoneal dialysis; HD: hemodialysis; ESRD: end stage renal disease; CGN: chronic glomerulonephritis; PKD: polycystic kidney; WBC: white blood cells; TG: triglyceride; TC: total cholesterol; HDL: high-density lipoprotein; LDL: low-density lipoprotein; Cr: creatinine; UA: uric acid; BUN: blood urea nitrogen; eGFR: estimated glomerular filtration rate; BG: blood glucose.

### The influences of BI on mechanical complications

The incidence of mechanical complications and the rates of surgical correction are shown in Table 2. There were no significant differences in mechanical complications (dialysate leakage, catheter migration, omental wrap, and hernia), or the need for surgical correction of these complications between the BI ≤ 24 h group and BI > 24 h group at each follow-up timepoint (*p* > 0.05) (Table 2).

**Table 2.** Mechanical and infectious complications between different BI in patients with urgent PD within varies follow-up time.

	Overall (*n* = 871)	BI ≤ 24h (*n* = 470)	BI > 24h (*n* = 401)	*X*^2^-value	*p*-Value
Mechanical complications					
Within 2 weeks [*n* (%)]					
Leakage	13 (1.5%)	8 (1.7%)	5 (1.2%)	0.305	0.581
Migration	52 (6.0%)	25 (5.3%)	27 (6.7%)	0.771	0.380
Omental wrap	2 (0.2%)	1 (0.2%)	1 (0.2%)	–	1.000
Requiring surgical correction [*n* (%)]					
Migration	11 (1.3%)	9 (1.9%)	2 (0.5%)	3.480	0.062
Omental wrap	2 (0.2%)	1 (0.2%)	1 (0.2%)	–	1.000
Within 1 month [*n* (%)]					
Leakage	13 (1.5%)	8 (1.7%)	5 (1.2%)	0.305	0.581
Migration	56 (6.4%)	25 (5.3%)	31 (7.7%)	2.092	0.148
Omental wrap	4 (0.5%)	3 (0.6%)	1 (0.2%)	0.118	0.731
Requiring surgical correction [*n* (%)]					
Migration	12 (1.4%)	9 (1.9%)	3 (0.7%)	2.168	0.141
Omental wrap	4 (0.5%)	3 (0.6%)	1 (0.2%)	0.118	0.731
Within 3 months [*n* (%)]					
Leakage	13 (1.5%)	8 (1.7%)	5 (1.2%)	0.305	0.581
Hernia	1 (0.1%)	0 (0.0%)	1 (0.2%)	–	0.460
Migration	65 (7.5%)	30 (6.4%)	35 (8.7%)	1.723	0.189
Omental wrap	5 (0.6%)	4 (0.9%)	1 (0.2%)	0.521	0.471
Requiring surgical correction [*n* (%)]					
Hernia	1 (0.1%)	0 (0.0%)	1 (0.2%)	–	0.460
Migration	15 (1.7%)	11 (2.3%)	4 (1.0%)	2.306	0.129
Omental wrap	5 (0.6%)	4 (0.9%)	1 (0.2%)	0.521	0.471
Within 6 months [*n* (%)]					
Leakage	15 (1.7%)	10 (2.1%)	5 (1.2%)	0.992	0.319
Hernia	3 (0.3%)	1 (0.2%)	2 (0.5%)	0.019	0.890
Migration	68 (7.8%)	32 (6.8%)	36 (9.0%)	1.414	0.234
Omental wrap	5 (0.6%)	4 (0.9%)	1 (0.2%)	0.521	0.471
Requiring surgical correction [*n* (%)]					
Hernia	2 (0.2%)	0 (0.0%)	2 (0.5%)	–	0.212
Migration	17 (2.0%)	12 (2.6%)	5 (1.2%)	1.930	0.165
Omental wrap	5 (0.6%)	4 (0.9%)	1 (0.2%)	0.521	0.471
Infectious complications					
Within 2 weeks [*n* (%)]	33 (3.8%)	18 (3.8%)	15 (3.7%)	0.005	0.945
Within 1 month [*n* (%)]	50 (5.7%)	28 (6.0%)	22 (5.5%)	0.089	0.766
Within 3 months [*n* (%)]	92 (10.6%)	46 (9.8%)	46 (11.5%)	0.650	0.420
Within 6 months [*n* (%)]	122 (14.0%)	59 (12.6%)	63 (15.7%)	1.791	0.181

BI: break-in period.

**Figure 2.**
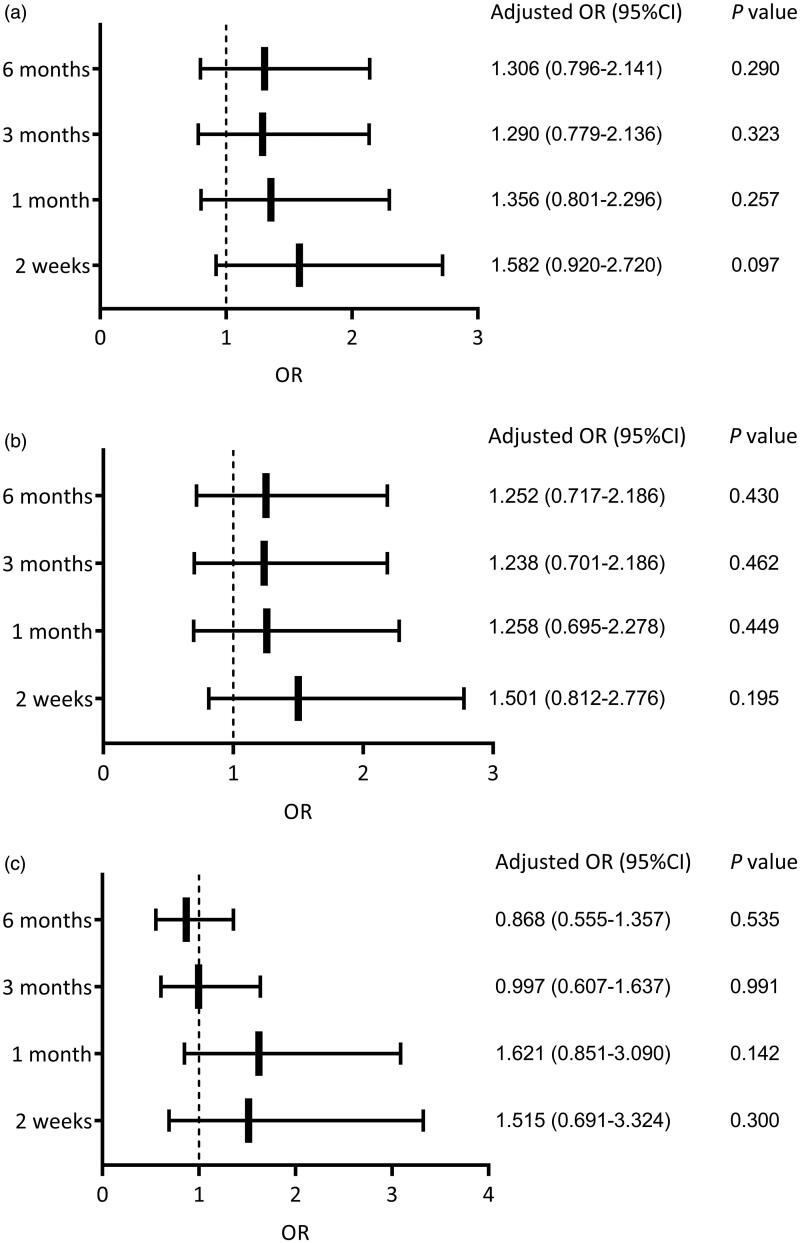
Logistic multivariate analyses at different follow-up timepoints. (a) The influence of BI on mechanical complications, adjusted for center, sex, age, temporary HD usage, history of abdominal surgery, dialysis mode during the BI period, combined diabetes, and WBC count. (b) The influence of BI on catheter migration, adjusted for center, sex, age, temporary HD usage, dialysis mode during the BI period, cause of ESRD, combined diabetes, history of abdominal surgery, and WBC count. (c) The influence of BI on infectious complications, adjusted for center, sex, age, temporary HD usage, dialysis mode during the BI period, combined hypertension, hemoglobin, albumin, HDL, LDL and BG. BI: break-in period; HD: hemodialysis; WBC: white blood cells; ESRD: end-stage renal disease; HDL: high-density lipoprotein; LDL: low-density lipoprotein; BG: blood glucose; OR: odds ratio; CI: confidence interval.

After adjustment for center, sex, age, temporary HD usage, history of abdominal surgery, dialysis mode during the BI period, combined diabetes, and WBC count, we found that BI ≤ 24 h was not an independent risk factor for mechanical complications at each follow-up timepoint (*p* > 0.05) (Figure 2(a)). Likewise, BI ≤ 24 h was not an independent risk factor for catheter migration after adjustment for center, sex, age, temporary HD usage, dialysis mode during the BI period, cause of ESRD, combined diabetes, history of abdominal surgery, and WBC count (*p* > 0.05) (Figure 2(b)).

**Figure 3.**
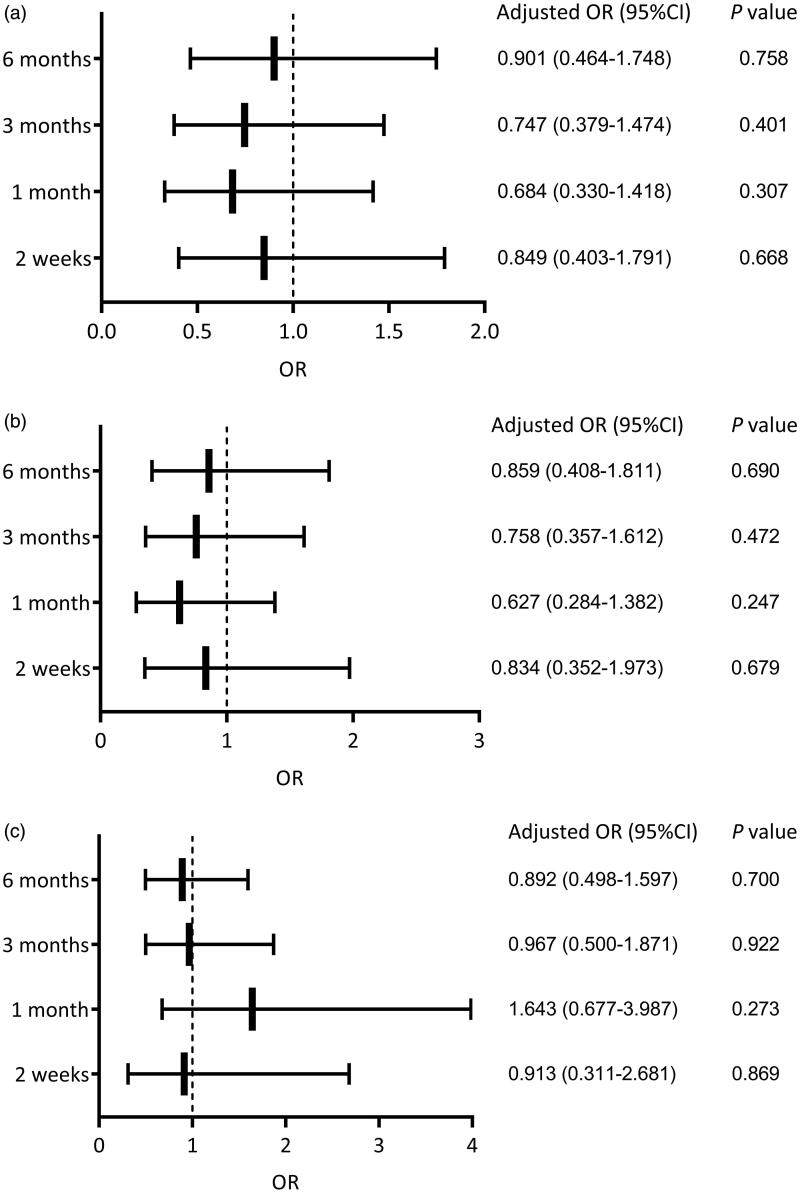
Logistic multivariate analyses at different follow-up timepoints for the patients who did not receive temporary HD. (a) The influence of BI on mechanical complications, adjusted for center, sex, age, dialysis mode during the BI period, history of abdominal surgery, combined diabetes, WBC count, HDL, UA, and Na. (b) The influence of BI on catheter migration, adjusted for center, sex, age, dialysis mode during the BI period, cause of ESRD, history of abdominal surgery, combined diabetes, WBC count, HDL and Na. (c) The influence of BI on infectious complications, adjusted for center, sex, age, dialysis mode during the BI period, history of abdominal surgery, albumin and LDL. HD: hemodialysis; BI: break-in period; WBC: white blood cells; HDL: high-density lipoprotein; UA: uric acid; ESRD: end-stage renal disease; LDL: low-density lipoprotein; OR: odds ratio; CI: confidence interval.

For patients who did not receive temporary HD, BI ≤ 24 h was not an independent risk factor for mechanical complications at each follow-up timepoint after adjustment for center, sex, age, dialysis mode during the BI period, history of abdominal surgery, combined diabetes, WBC count, HDL, UA, and Na (*p* > 0.05) (Figure 3(a)). Similarly, we found a BI ≤ 24 h was not an independent risk factor for catheter migration after adjustment for center, sex, age, dialysis mode during the BI period, cause of ESRD, history of abdominal surgery, combined diabetes, WBC count, HDL and Na (*p* > 0.05) (Figure 3(b)).

### The influences of BI on infectious complications

The pooled rate of infectious complications at each follow-up timepoint are shown in Table 2. There were no significant differences in infectious complications between the two groups at each follow-up timepoint (*p* > 0.05).

A BI ≤ 24 h was not a significant risk factor for infectious complications at each follow-up timepoint after adjustment for center, sex, age, temporary HD usage, dialysis mode during the BI period, combined hypertension, hemoglobin, albumin, HDL, LDL and BG (*p* > 0.05) (Figure 2(c)).

For patients who did not receive temporary HD, a BI ≤ 24 h was not an independent risk factor for infectious complications after adjustment for center, sex, age, dialysis mode during the BI period, history of abdominal surgery, albumin, and LDL (*p* > 0.05) (Figure 3(c)).

### The influences of BI on technique failure

Using multivariate Cox regression analysis, we found that BI ≤24 h was not an independent risk factor for technique failure after adjustment for center, sex, age, dialysis mode during the BI period, triglyceride, TC, Na, and BG (OR = 0.857, 95%CI = 0.253–2.902, *p* > 0.05).

A sub-analysis of patients who did not receive temporary HD, using multivariate Cox regression analysis, found that a BI ≤24 h was not an independent risk factor for technique failure after adjustment for center, sex, age, dialysis mode during the BI period, WBC count, triglyceride, TC, LDL, BUN, Na, and BG (OR = 1.657, 95%CI = 0.295–9.312, *p* > 0.05).

### The influences of dialysis mode during the BI period on dialysis-related complications and technique failure

After adjustment for potential confounders, APD during the BI period was an independent protective factor for infectious complications at 2 weeks (*p* < 0.05), but not at 1 month, 3 months, or 6 months (*p* > 0.05) (Supplementary Figure 1(c)). Nevertheless, for mechanical complications (Supplementary Figure 1(a,b)) and technique failure (Supplementary Figure 3), APD during the BI period was not an independent protective factor after adjustment for potential confounders (*p* > 0.05). Of note, for patients who did not receive temporary HD, APD during the BI period was not an independent risk factor for mechanical complications (Supplementary Figure 2(a,b)), infectious complications (Supplementary Figure 2(c)), and technique failure (Supplementary Figure 3) (*p* < 0.05).

## Discussion

To the best of our knowledge, this is the first multicenter study to investigate the feasibility of a BI ≤ 24 h in patients requiring USPD. Our study demonstrated that initiating PD within 24 h of catheter insertion had no major influence on mechanical complications, infectious complications, or technique survival.

We focused on the influence of a BI ≤ 24 h on the incidence of PD-related complications (especially mechanical complications) when compared with a BI > 24 h. Two studies which assessed a BI < 24 h from Korea with a follow-up of 1 month found that the incidence of catheter migration was 8.5% and 6.0%, respectively, and the incidence of catheter leakage was 10.2% and 2.0%, respectively. Unfortunately, neither of these studies had a control group [19,20]. Another study with a follow-up of 3 months reported that for a BI < 24 h and a BI > 7 days the incidence of mechanical complications was 9.7% and 5.4%, respectively. Although the difference was not statistically significant, percutaneous catheter surgery was more commonly used for a BI < 24 h and laparoscopic or open surgery method was more commonly used for a BI > 7 days. Thus, the surgical modality may have affected the results [22]. A study from Denmark found that the incidences of mechanical complications and surgical corrections were significantly higher with a BI < 24 h than a BI > 12 days at 3 months follow-up [23]. However, there were several issues in this article that should not be ignored: (1) the study had a small sample size (52 patients with USPD vs 88 patients with planned PD); (2) the study excluded patients with signs of uremic pericarditis, severe volume overload or pulmonary edema, severe hypertension (diastolic blood pressure > 120 mmHg), severe hyperkalemia (K^+^>6.5 mmol/L), or colitis, as these conditions were considered to be contraindications for USPD; and (3) the dialysis modality was limited to APD. Our results differ from those of that study, which may be because all the above issues were addressed in our study.

We found no significant differences in mechanical complications between a BI ≤ 24 h and a BI > 24 h. Our results are more reliable than previous studies for the following additional reasons: (1) the data was collected from multiple centers, which makes this a more representative cohort; (2) this was a real-world multicenter retrospective cohort study, and we retained the data about temporary HD (some of the patients had to undergo temporary HD because open surgery could not be performed immediately) and patients with urgent dialysis indications (such as severe volume overload or pulmonary edema, and severe hyperkalemia [K^+^>6.5 mmol/L]); (3) both APD and CAPD were used as initiating dialysis modalities in our study; and (4) The same type of procedure was performed for each patient. Currently, much controversy exists regarding which is the optimal surgical procedure for PD. Some studies have reported that a percutaneous or laparoscopic approach, rather than open surgery, is optimal regarding complications and catheter survival [32–34]. However, other studies have reported no significant relationship between surgical methods and complications [35,36]. To avoid the impact of surgical modality on the results, only patients who received open surgery were included in our analysis.

Previous studies have not agreed on whether a BI < 24 h is a risk factor for mechanical complications [19–23]. Our study found that a BI ≤ 24 h was not an independent risk factor for mechanical complications or catheter migration after considering the differences in BI, dialysis mode during the BI period, and perioperative management at the five centers in our study, and adjusting for the center as a variable. One possible reason for this is that although PD was initiated within 24 h of catheter insertion, the incremental initiation of PD treatment led to a piecemeal increase in patient intra-abdominal pressure and decreased flotation of the catheter, reducing mechanical complications.

Infectious complications are one of the main causes of technique failure, accounting for 52% of cases [37]. Therefore, it is important to investigate the infectious complications of USPD. A previous study suggested that APD decreases the incidence of peritonitis by reducing the number of connections and disconnections per day compared to CAPD [38]. Similarly, our results demonstrated that APD was an independent protective factor for infectious complications at the 2 weeks follow-up. However, this phenomenon was no longer present at the 1-month follow-up, which may be associated with improvements in the patient’s ability to successfully perform CAPD in a sterile manner. In addition, APD was not related to the infectious complications at 2 weeks in the analysis where patients who received temporary HD were excluded. Fourteen patients who received temporary HD had infectious complications at the 2 week follow up. Among these were four patients with APD and 10 patients with CAPD (data not shown), which indicated that temporary HD may be associated with infectious complications.

A study using only APD as the initiating dialysis modality revealed no difference in infectious complications between a BI < 24 h and a BI > 12 days [23]. Using multiple logistic regression for our data, we calculated that a BI ≤ 24 h was not a significant risk factor for infectious complications when compared with a BI > 24 h after adjusting for confounding factors. In addition, as the use of HD catheters would also affect infection rates, we could still conclude that a BI ≤ 24 h was not a significant risk factor for infectious complications for patients who did not receive temporary HD, after adjustment of confounding factors. We believe our findings may also be more reliable as both APD and CAPD were used to initiate dialysis. All five centers included in our study had extensive experience with PD. Therefore, the incidence of infectious complications could also have been reduced by improved aseptic operation and patient education, as supported by the findings of report by Figueiredo et al. [39].

The high incidence of technique failure has been recognized as a critical factor in the relatively poor PD retention rates worldwide [40]. The technique survival rate with a BI < 24 h was 95% according to a report from Korea with a 1 month follow-up period [20] and was 97.8%, according to another report from Italy with a 3 month follow-up period [21]. Povlsen et al. reported that technique survival for a BI < 24 h and BI ≥ 12 days at 3 months were 75% and 86.5%, respectively, with no significant difference between the two groups [23]. This is similar to our findings. Logistic regression demonstrated that a BI ≤ 24 h was not an independent risk factor for technique failure. This could be explained by findings of a previous study which reported that larger dialysis centers and centers with more PD patients had a lower risk of technique failure and that patient race also affects technique failure, with a lower incidence of technique rejection seen in Asian patients [37]. In this study, all procedures were performed at large dialysis centers and all patients included in our study were Asian.

There are several limitations to this study. First, patients were screened by retrospective analysis rather than randomization, which may have introduced a selection bias to this study. Second, due to incomplete data, some potentially important data such as dialysis adequacy were not used as primary endpoints. Third, the data were all from Northeast China, and our results may not be generalizable to other areas of the world.

## Conclusion

Overall, our results suggest that a BI of less than 24 h did not increase the incidence of mechanical complications, infectious complications, or technique failure compared with a BI of more than 24 h.

## Supplementary Material

Supplemental Material

Supplemental Material

Supplemental Material

Supplemental Material

Supplemental Material

Supplemental Material

Supplemental Material

Supplemental Material

## Data Availability

The data supporting the findings of this study may be provided by the corresponding author upon reasonable request.

## Geolocation information

The Second Hospital of Jilin University, No. 218 Ziqiang Street, Nanguan District, Changchun 130041, China.
