# Supplementary figures and images for: Feasibility of a break-in period of less than 24 hours for urgent start peritoneal dialysis: a multicenter study
Source: Ren Fail. 2022 Mar 10;44(1):450–60. doi: 10.1080/0886022X.2022.2049306 (PMC8920377; doi:10.1080/0886022X.2022.2049306)

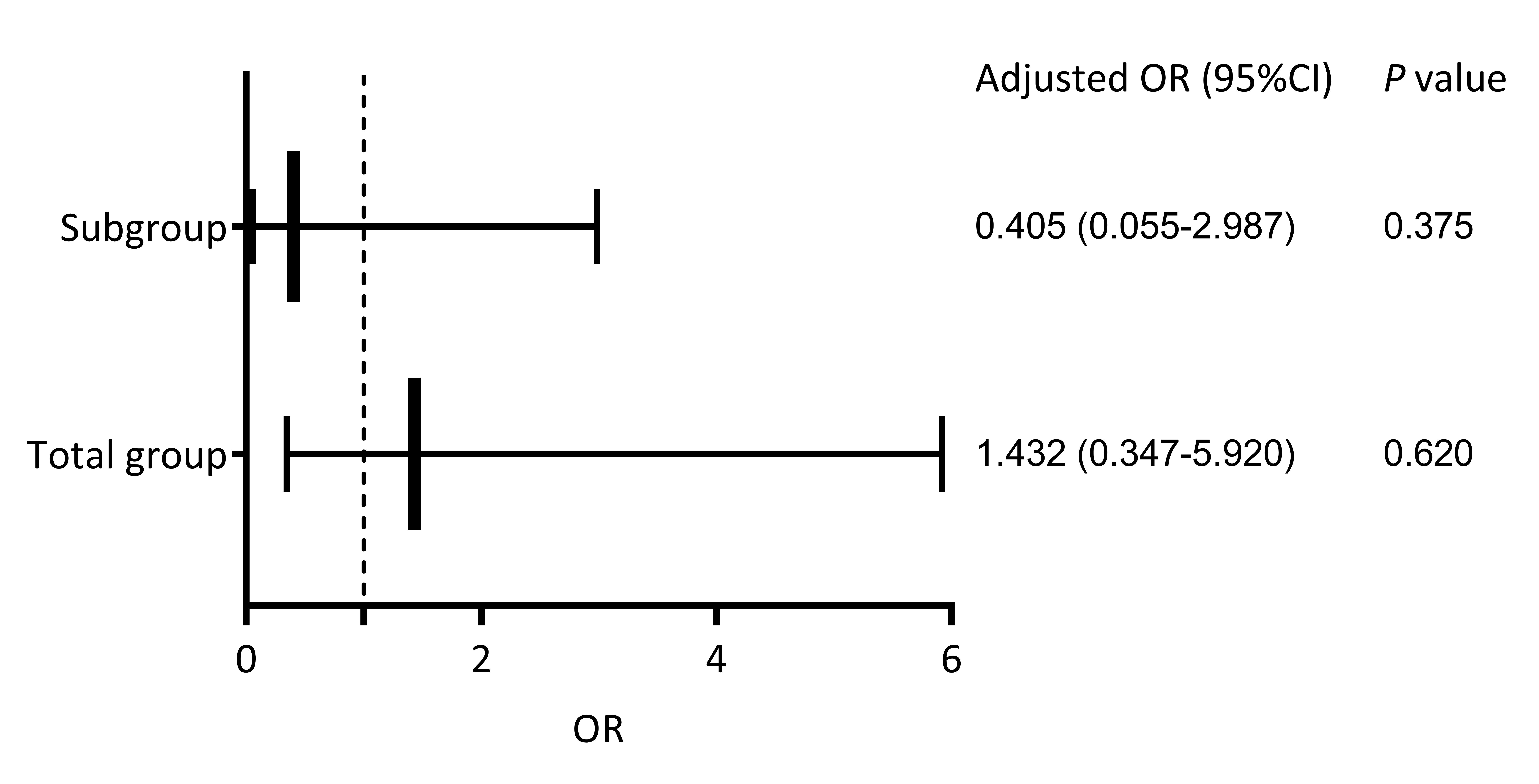

Supplement: Supplemental Material [file IRNF_A_2049306_SM0484.jpg]

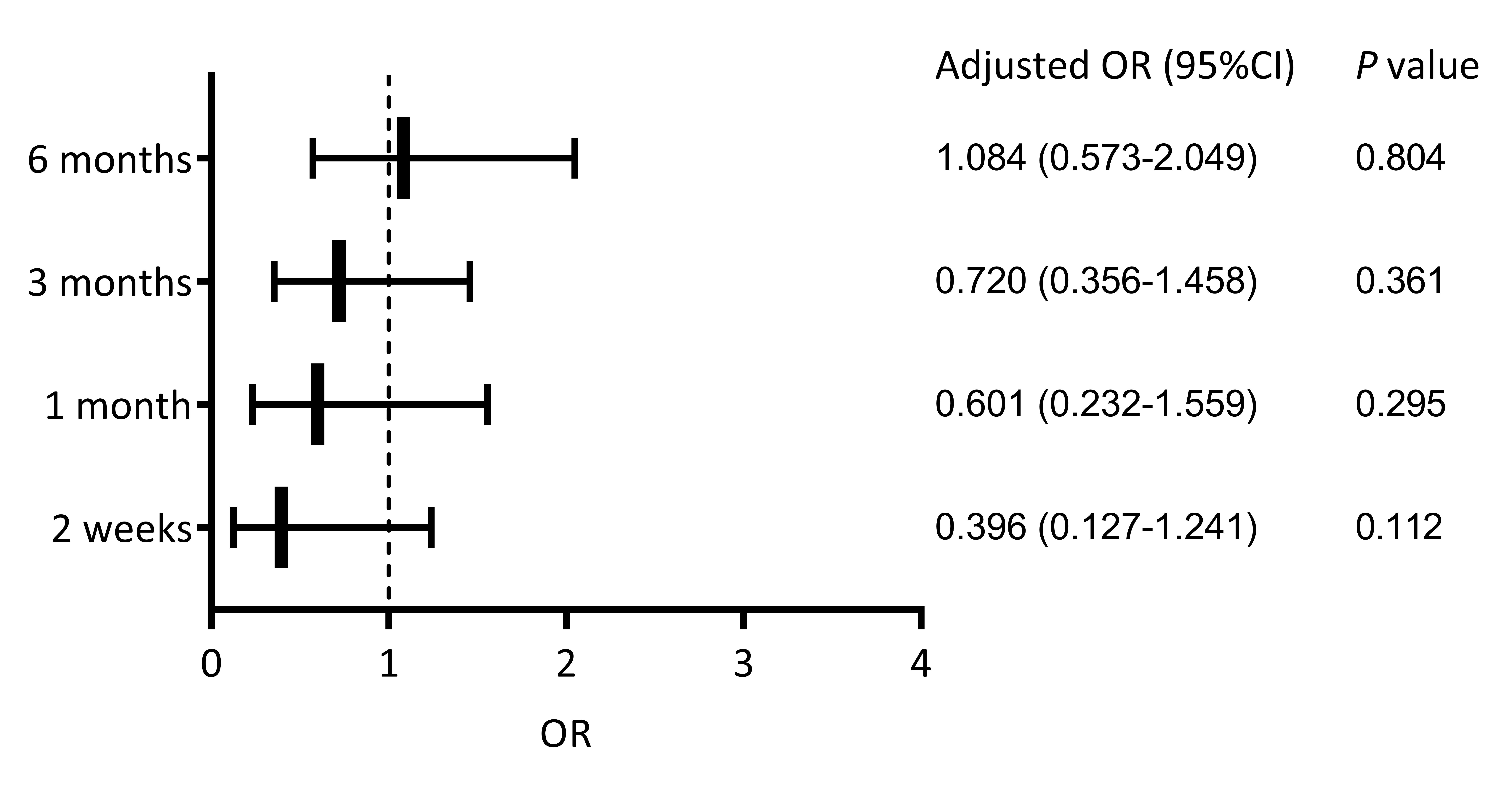

Supplement: Supplemental Material [file IRNF_A_2049306_SM0463.jpg]

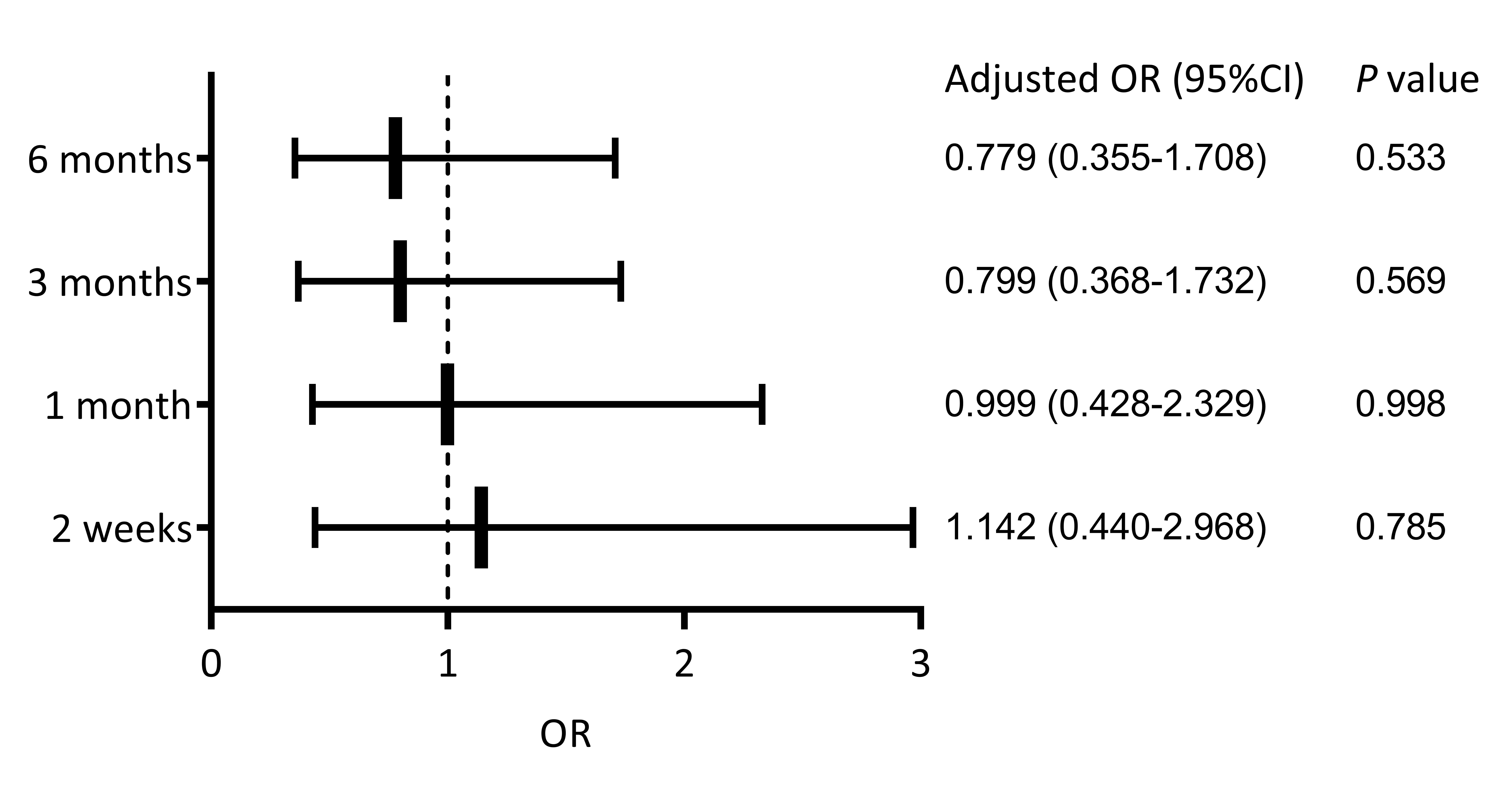

Supplement: Supplemental Material [file IRNF_A_2049306_SM0450.jpg]

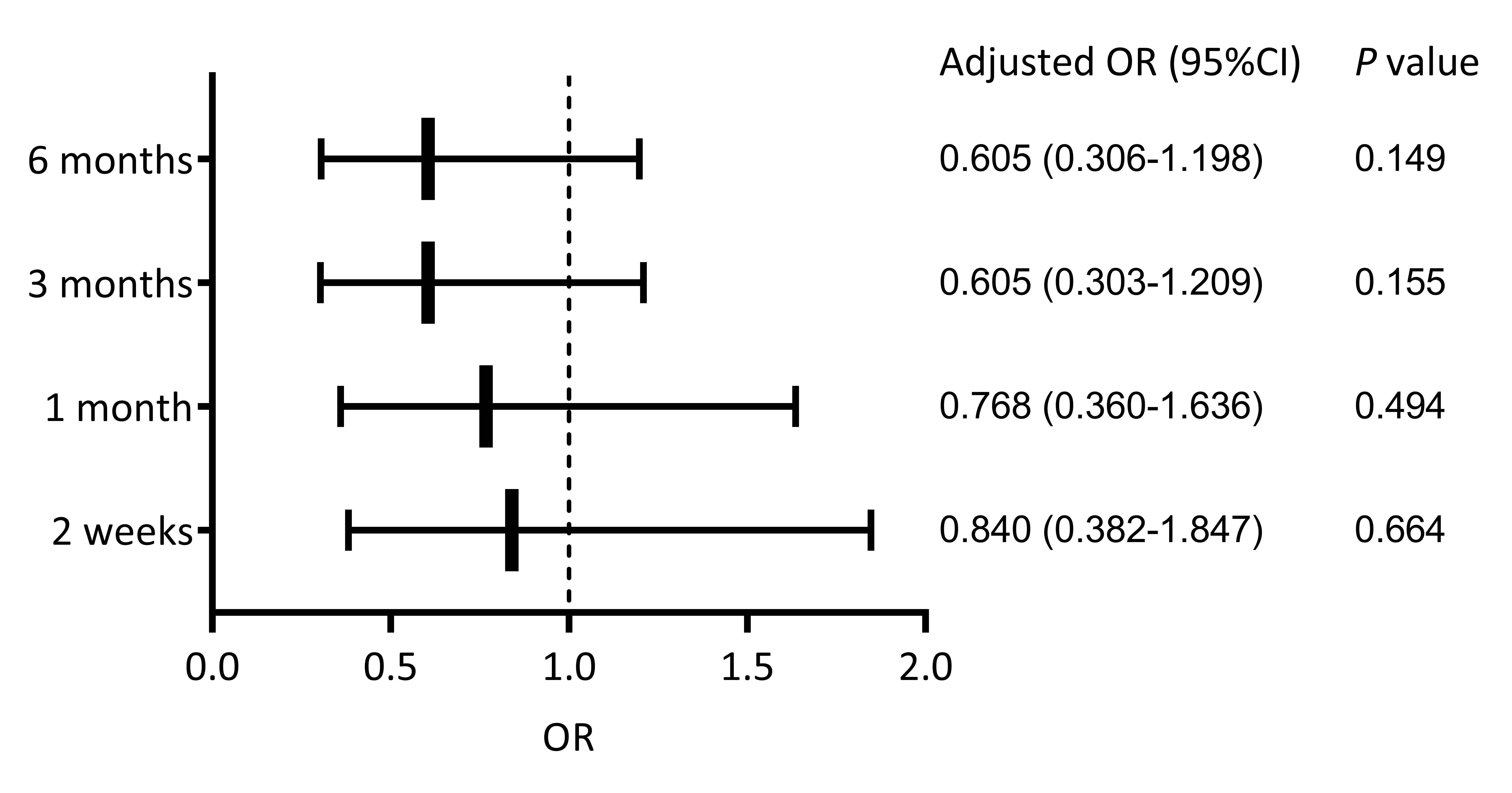

Supplement: Supplemental Material [file IRNF_A_2049306_SM0445.jpg]

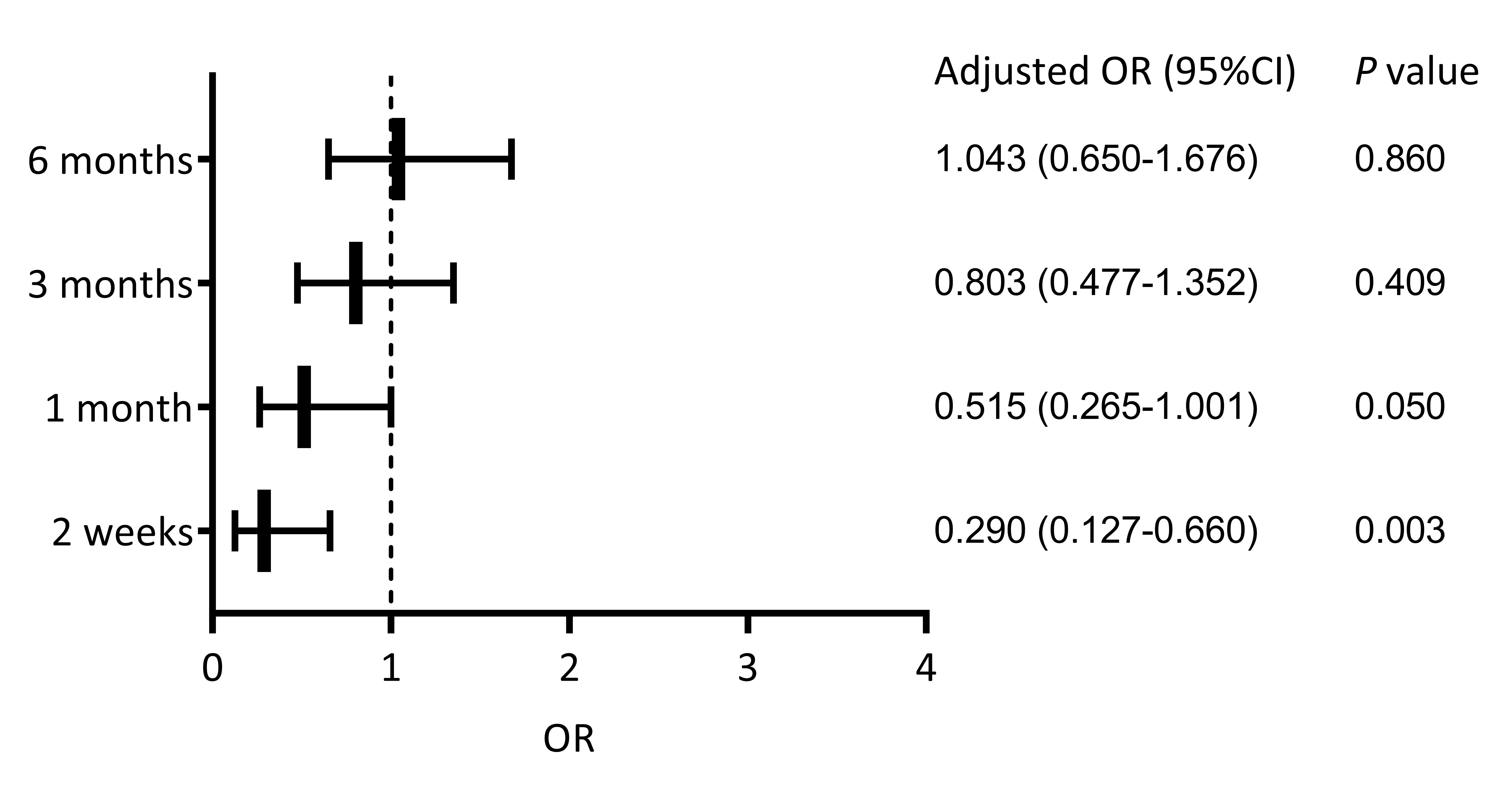

Supplement: Supplemental Material [file IRNF_A_2049306_SM0359.jpg]

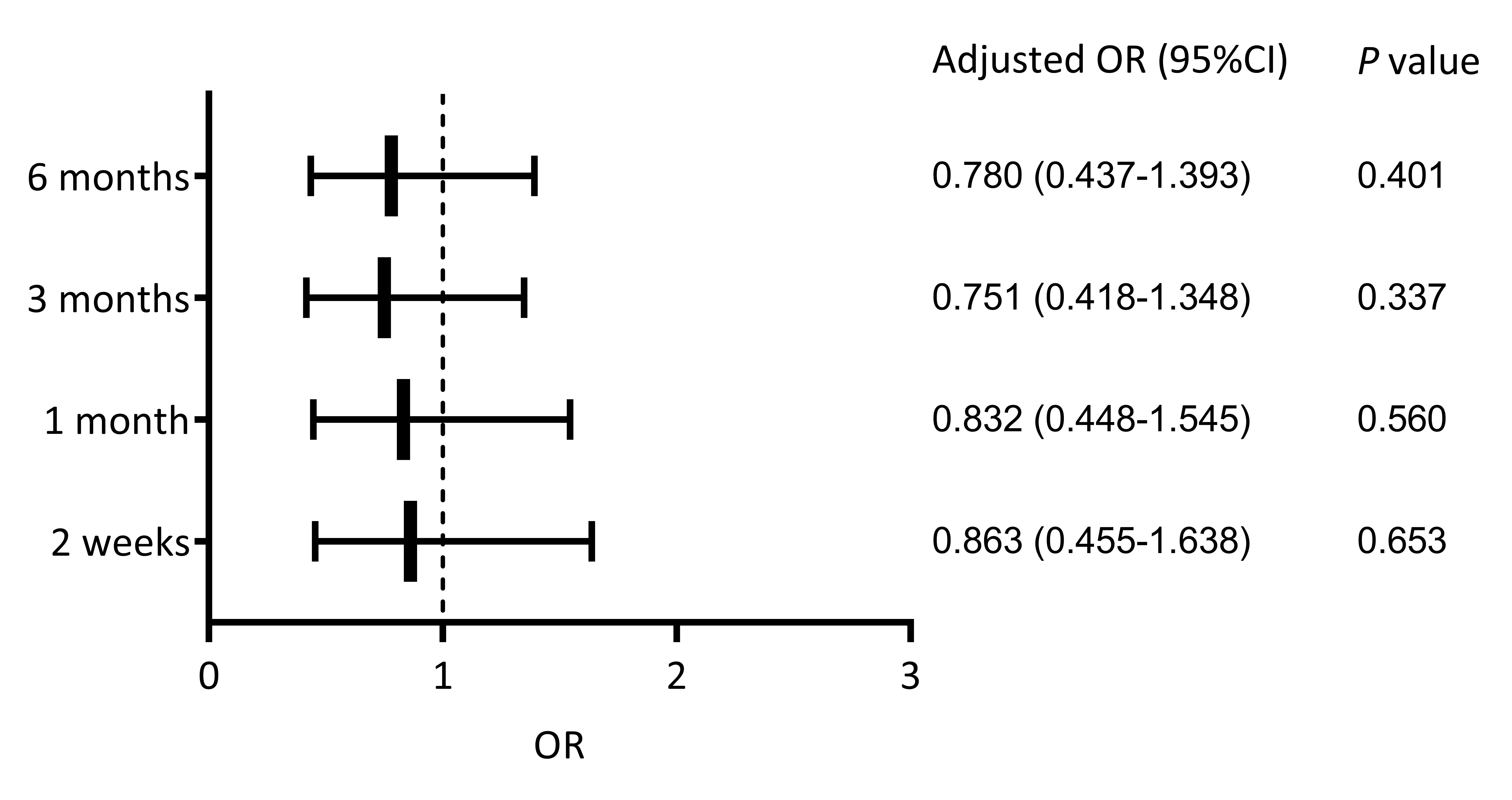

Supplement: Supplemental Material [file IRNF_A_2049306_SM0316.jpg]

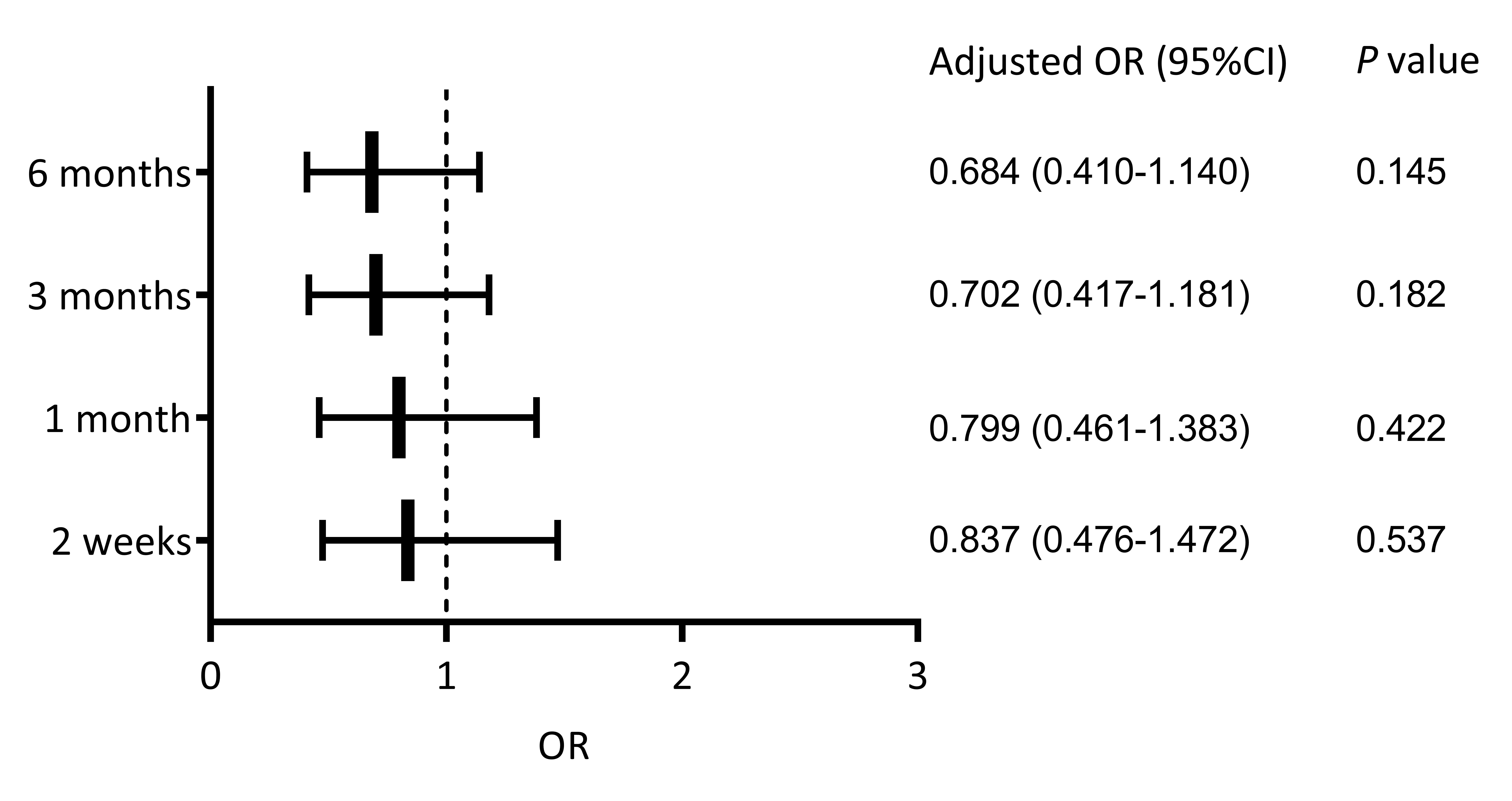

Supplement: Supplemental Material [file IRNF_A_2049306_SM0305.jpg]
