# Supplementary material for: Feasibility of a break-in period of less than 24 hours for urgent start peritoneal dialysis: a multicenter study
Source: Ren Fail. 2022 Mar 10;44(1):450–60. doi: 10.1080/0886022X.2022.2049306 (PMC8920377; doi:10.1080/0886022X.2022.2049306)
Supplement: Supplemental Material [file IRNF_A_2049306_SM0289.pdf]

Supplemental Figure1. Logistic multivariate analyses at different follow-up timepoints. Figure 1a The influence of APD during the BI period on mechanical complications, adjusted for center, sex, age, BI, temporary HD usage, history of abdominal surgery, combined diabetes, and WBC count. Figure 1b The influence of APD during the BI period on catheter migration, adjusted for center, sex, age, BI, temporary HD usage, cause of ESRD, combined diabetes, history of abdominal surgery, and WBC count. Figure 1c The influence of APD during the BI period on infectious complications, adjusted for center, sex, age, BI, temporary HD usage, combined hypertension, hemoglobin, albumin, HDL, LDL and BG.

APD: automated peritoneal dialysis; BI: break-in period; HD: hemodialysis; WBC: white blood cells; ESRD: end-stage renal disease; HDL: high-density lipoprotein; LDL: low-density lipoprotein; BG: blood glucose; OR: odds ratio; CI: confidence interval.

Supplemental Figure 2. Logistic multivariate analyses at different follow-up timepoints for the patients who did not receive temporary HD. Figure 2a The influence of APD during the BI period on mechanical complications, adjusted for center, sex, age, BI, history of abdominal surgery, combined diabetes, WBC count, HDL, UA, and Na. Figure 2b The influence of APD during the BI period on catheter migration, adjusted for center, sex, age, BI, cause of ESRD, history of abdominal surgery, combined diabetes, WBC count, HDL and Na. Figure 2c The influence of APD during the BI period on infectious complications, adjusted for center, sex, age, BI, history of abdominal surgery, albumin and LDL.

HD: hemodialysis; APD: automated peritoneal dialysis; BI: break-in period; WBC: white blood cells; HDL: high-density lipoprotein; UA: uric acid; ESRD: end-stage renal disease; LDL: low-density lipoprotein; OR: odds ratio; CI: confidence interval.

Supplemental Figure 3. Multivariate Cox modeling analysis for patients. The influence of APD during the BI period on technique failure for total group and subgroup. Total group adjusted for center, sex, age, BI, triglyceride, TC, Na, and BG. Subgroup adjusted

for center, sex, age, BI, WBC count, triglyceride, TC, LDL, BUN, Na, and BG.

APD: automated peritoneal dialysis; BI: break-in period; TC: total cholesterol; BG: blood glucose; WBC: white blood cells; LDL: low-density lipoprotein; BUN: blood urea nitrogen; OR: odds ratio; CI: confidence interval.
